# Do men with prostate or colorectal cancer seek different information and support from women with cancer?

**DOI:** 10.1054/bjoc.2001.1945

**Published:** 2001-09

**Authors:** M Boudioni, K McPherson, C Moynihan, J Melia, M Boulton, G Leydon, J Mossman

**Affiliations:** Cancer BACUP, 3 Bath Place, Rivington Street, London, EC2 3JR; Cancer and Public Health Unit, London School of Hygiene and Tropical Medicine, Keppel Street, London, WC1E 7HT; Institute of Cancer Research and the Royal Marsden NHS Trust, Downs Road, Sutton, SM2 5PT; Cancer Screening Evaluation Unit, Institute of Cancer Research, 15 Cotswold Road, Sutton, SM2 5NG; School of Social Sciences and Law, Oxford Brookes University, Gipsy Lane Campus, Oxford, OX3 0BP, UK

**Keywords:** cancer patients, men, information and support requests, colorectal cancer, prostate cancer

## Abstract

Male cancer patients' use of a national cancer information service, their requests and key predictors of these over the period April 1996 to March 1998 are presented, in comparison with women. The most frequent requests of 411 prostate, 162 male and 217 female colorectal cancer patients were similar: site-specific information, emotional support, publications, specific therapies. Research or clinical trials (*P*< 0.05), diet and nutrition (*P*< 0.001) requests differed between men with prostate and colorectal cancers; complementary therapies (*P*< 0.05), prognosis (*P*< 0.05) requests differed between male and female colorectal cancer patients. Among prostate cancer patients, employed men aged 60+ were more likely to need emotional support than retired men aged 70 +; men < 59 years old were more likely to request publications, but less likely to enquire about specific therapies than others. Among male colorectal cancer patients, employed men were less likely to request site-specific information, but more likely to need emotional support than retired men; patients from geographical areas other than Thames were more likely to request publications; patients from manual classes were less likely to enquire about specific therapies than those from non-manual classes. The complexity of information and support seeking behaviour is demonstrated; no pattern was found among men or in comparison with women. Further research is needed to enable development of services that are appropriate to individual needs and concerns. © 2001 Cancer Research Campaign http://www.bjcancer.com


*British Journal of Cancer* (2001) **85**(5), 641–648
© 2001 Cancer Research Campaign
doi: 10.1054/ bjoc.2001.1945, available online at http://www.idealibrary.com on IDEAL

http://www.bjcancer.com

### Do men with prostate or colorectal cancer seek different information and support from women with cancer?

**M Boudioni[1], K McPherson[2], C Moynihan[3], J Melia[4], M Boulton[5], G Leydon[2] and J Mossman[1]**

[1]CancerBACUP, 3 Bath Place, Rivington Street, London EC2 3JR; [2]Cancer and Public Health Unit, London School of Hygiene and Tropical Medicine, Keppel Street, London WC1E 7HT; [3]Institute of Cancer Research and the Royal Marsden NHS Trust, Downs Road, Sutton SM2 5PT; [4]Cancer Screening Evaluation Unit, Institute of Cancer Research, 15 Cotswold Road, Sutton SM2 5NG; and [5]School of Social Sciences and Law, Oxford Brookes University, Gipsy Lane Campus, Oxford OX3 0BP, UK

**Summary** Male cancer patients' use of a national cancer information service, their requests and key predictors of these over the period April 1996 to March 1998 are presented, in comparison with women. The most frequent requests of 411 prostate, 162 male and 217 female colorectal cancer patients were similar: site-specific information, emotional support, publications, specific therapies. Research or clinical trials ($P$ < 0.05), diet and nutrition ($P$ < 0.001) requests differed between men with prostate and colorectal cancers; complementary therapies ($P$ < 0.05), prognosis ($P$ < 0.05) requests differed between male and female colorectal cancer patients. Among prostate cancer patients, employed men aged 60+ were more likely to need emotional support than retired men aged 70 +; men < 59 years old were more likely to request publications, but less likely to enquire about specific therapies than others. Among male colorectal cancer patients, employed men were less likely to request site-specific information, but more likely to need emotional support than retired men; patients from geographical areas other than Thames were more likely to request publications; patients from manual classes were less likely to enquire about specific therapies than those from non-manual classes. The complexity of information and support seeking behaviour is demonstrated; no pattern was found among men or in comparison with women. Further research is needed to enable development of services that are appropriate to individual needs and concerns. © 2001 Cancer Research Campaign http://www.bjcancer.com

**Keywords**: cancer patients; men; information and support requests; colorectal cancer; prostate cancer

Cancer is diagnosed as frequently in men as in women. In England and Wales in 1994 the female to male incidence ratio, excluding non-melanoma skin cancer, was 1:1 (ONS, 2000). However, studies have demonstrated that men are low users of cancer information services, as well as other health and social support services (Slevin et al, 1988; Greenglass, 1992; Manfredi et al, 1993; Harrison et al, 1995; Boudioni et al, 1999a; Green and Pope, 1999; Williams et al, 1999). About 60% of patients contacting the National Cancer Information Service in the USA in 1993 were female (Manfredi et al, 1993). Similarly, 80% of the CancerBACUP Information Service users in its first 2 years were women (Slevin et al, 1988). More recently, Boudioni et al (1999a) found an excess of both female enquirers and female patients enquired about among the service's first time users in a 1-year period, compared with the Great Britain population and cancer incidence in women respectively (SIRs = 1.51 and 1.18).

Other research has shown that men are not 'health aware' (Kirby and Kirby, 1999), and are far more reluctant than women to monitor their health and seek professional help at an early stage (Health Education Authority, 1996). There is also a high level of 'ignorance' amongst men about male-specific cancers, such as prostate and testicular (MORI, 1999), partly because the information is not available to them (MORI, 1999). On the other hand, the utility of information and support for cancer patients, regardless of gender, has been well documented (Audit Commission, 1993; Expert Advisory Group, 1995; Fallowfield et al, 1995; National Cancer Alliance, 1996; Leydon et al, 2000), and there are no data to suggest that men benefit less than women from support and accurate, up-to-date information.

The government and the National Cancer Director have committed to equality of care and access to everyone (Expert Advisory Group, 1995; DoH, 1997; DoH, 1998; DoH, 1999; DoH, 2000a) with the patient at the heart of the health service (The Stationery Office, 2000). The health of men has been identified as a topic of special interest (DoH, 1992). The Men's Health Forum (1997) has been calling for awareness and promotion of men's health since 1994. The Everyman Campaign – launched by the Institute of Cancer Research – has been raising awareness about prostate and testicular cancer since 1997 (MORI, 1999). Although less money has been spent on men's health than on women's, the Public Health Minister has recently announced increased funding for research on prostate cancer (DoH, 2000b).

In the light of such interest, this study was designed to examine men's health behaviour with respect to their information and support-seeking patterns. It compares men with different types of cancer (prostate and colorectal), and men and women with the same type of cancer (colorectal), who used a cancer information service. Prostate and colorectal cancers are not only among the most common cancers for men nationally, but they were also the two cancers most frequently enquired about by men with cancer

*Received 12 October 2000*
*Revised 9 May 2001*
*Accepted 1 June 2001*

*Correspondence to:* M Boudioni

BJOC 01-1945 641-648 20/8/01 3:11 pm Page 642

contacting the CancerBACUP Information Service over the period April 1996 to March 1998. Breast, or another female cancer, was not selected for comparison, because of the differences in illness and treatment characteristics, publicity and awareness about the specific cancer. The main aims of the study were to describe male cancer patients' use of a national cancer information service, their information and support requests, and key predictors of these requests. A better understanding of the health behaviour of men with cancer, and those with prostate and colorectal cancers, in particular, is important for the development of information and support services that are appropriate to their needs and concerns.

### SUBJECTS AND METHODS

### Sampling method and data collection

An Enquirer Record Form is systematically completed for every fifth enquirer to the CancerBACUP Information Service (Boudioni et al, 1999a). An extensive coding system with 64 codes is used to record requests for information and support. A maximum of six subjects of enquiry may be recorded for every enquirer. The forms are checked thoroughly and coded; they are then entered onto the database, currently Paradox, version 5.0.

Data collected from diagnosed patients between April 1996 and March 1998 were analysed for the purposes of this study. Data for each year are kept in separate database files; the files for the years April 1996 to March 1997 and April 1997 to March 1998 were merged. Since the Enquirer Record Form is updated each year, there were some changes in the coding system, especially in the 'subject of enquiry' fields. These were taken into account when merging the data.

Enquiries originating from outside the UK were excluded, thus only British data are used. The collection of data was good and there were only a few unknowns for most variables, which were classed as missing data and are reported but excluded from the analysis. Ethnic group data were collected from August 1996 only; as there was a high percentage of unknowns and over 80% of those recorded were white British, ethnicity was not included in the analysis.

### Statistical analysis

The statistical software SPSS, version 9.0, was used for data merging and for all subsequent analysis. Descriptive statistics were used initially to describe the age, employment status, social class and geographic location, as NHS regions, of prostate, and male and female colorectal, cancer patients. The most frequent subjects of enquiry of these groups were studied by tabulations and chi-square tests.

Among these patients, 'site specific information', 'emotional support / narratives', 'publications' and 'specific therapies' were the subjects or groups of subjects most frequently enquired about. The subgroups of prostate, and male and female colorectal cancer patients, enquiring about each of the four subjects were compared with all prostate, male or female colorectal cancer patients, regardless of subject of enquiry, using the Observed / Expected and Standardised Enquiry Ratio method (SER).

Logistic regression analyses were conducted initially for all patients to examine if gender, primary site or interactions were predictors of the four main subjects examined. Initial logistic regressions were also conducted for colorectal cancers to examine the effect of gender.

Logistic regression analyses were then conducted for prostate, and male and female colorectal cancer patients, to predict the presence or absence of the above four most frequent subjects or groups of subjects based on the socio-demographic variables: age, employment status, social class and geographic location. Wales was excluded from colorectal cancer analysis because of the very small number of enquiries.

The logistic regression method used for male cancer patients was backward elimination with the Likelihood-Ratio criterion. Interaction terms when a relation was suspected were used (Altman, 1997). The significance level of 0.10 for removal was used with a chosen cut off level of $P = 0.05$ for the score statistic. Significance was assessed from the reduction in the goodness of fit of the model. For female colorectal cancer patients, the significant predictors for male colorectal cancer patients were fitted, adjusted for age, into a simple enter logistic regression model. The significance of the Log Likelihood Ratio statistics, Odds Ratios and their 95% Confidence Intervals are presented.

### RESULTS

Between April 1996 and March 1998, 83 440 enquiries were answered. Of these, 14 100 (16.9%) enquiries were from health professionals, students, the 'worried well' and others. The majority of enquirers represented diagnosed patients (29 370, 35.2%) and relatives and friends of patients (39 970, 47.9%).

Data on 5874 diagnosed patients were collected (one in five sample); data for 5558 diagnosed patients were entered onto the database; data for 316 patients were not entered because they were incomplete. The gender was unknown for 5 patients; of the rest, 4249 were women and 1304 were men. The female to male ratio was 3.23:1.

The two commonest cancers enquired about by male patients were prostate (411, 31.5%) and colorectal (162, 12.4%); these patients together with female colorectal cancer patients (217, 5.1% of all female cancer patients) comprised the study sample. Other main cancer sites enquired about by men included Non-Hodgkin's lymphoma (95, 7.3%) and lung (89, 6.8%). In contrast, the other main cancers enquired about by women were breast (2,502, 58.9%), ovary (265, 6.2%) and Non-Hodgkin's lymphoma (128, 3.0%).

### Socio-demographic characteristics of prostate and colorectal cancer patients (Table 1)

The majority of prostate cancer patients were aged 60 or over (84.9%) and retired (61.6%); among colorectal cancer patients 59.3% of men and 47.3% of women were in this age group and 43.3% of men and 34.4% of women were retired. In all three groups, most enquirers were in non-manual social classes and more than 30% were from the Thames region.

### Subjects of enquiry of prostate and colorectal cancer patients (Table 2)

The most frequent requests from prostate, and male and female colorectal cancer patients alike were for site-specific information, emotional support and reassurance, publications, information about specific therapies and treatment side effects; their order, however, varied by site and gender.

Compared with prostate cancer patients, male colorectal cancer patients enquired significantly more frequently about

 © 2001 Cancer Research Campaign

BJOC 01-1945 641-648 20/8/01 3:11 pm Page 643

**Table 1** Socio-demographic characteristics of prostate, male and female colorectal cancer patients

| Socio-demographic characteristics | Prostate cancer patients | | Male colorectal cancer patients | | Female colorectal cancer patients | |
|---|---|---|---|---|---|---|
| | *n* = 411 | % | *n* = 162 | % | *n* = 217 | % |
| **Age distribution** | | | | | | |
| < 49 | 7 | 1.8 | 15 | 10.0 | 52 | 25.6 |
| 50–59 yrs | 51 | 13.3 | 46 | 30.7 | 55 | 27.1 |
| 60–69 yrs | 196 | 51.2 | 47 | 31.3 | 63 | 31.0 |
| 70 + yrs | 129 | 33.7 | 42 | 28.0 | 33 | 16.3 |
| **Total** | **383** | **100.0** | **150** | **100.0** | **203** | **100.0** |
| *Missing* | *28* | | *12* | | *14* | |
| Employment status | | | | | | |
| Employed | 132 | 35.5 | 80 | 53.7 | 81 | 41.5 |
| Retired | 229 | 61.6 | 64 | 43.0 | 67 | 34.4 |
| Unemployed & Other | 11 | 3.0 | 5 | 3.3 | 47 | 24.1 |
| **Total** | **372** | **100.0** | **149** | **100.0** | **195** | **100.0** |
| *Missing* | *39* | | *23* | | *22* | |
| **Social class** | | | | | | |
| I | 60 | 16.2 | 18 | 12.1 | 7 | 3.6 |
| II | 119 | 32.2 | 51 | 34.2 | 66 | 33.8 |
| III(NM) | 41 | 11.1 | 22 | 14.8 | 44 | 22.6 |
| III(M) | 55 | 14.9 | 23 | 15.4 | 4 | 2.1 |
| IV & V | 21 | 5.7 | 8 | 5.4 | 17 | 8.7 |
| Unclassified | 74 | 20.0 | 27 | 18.1 | 57 | 29.2 |
| **Total** | **370** | **100.0** | **149** | **100.0** | **195** | **100.0** |
| *Missing* | *41* | | *13* | | *22* | |
| **Geographic distribution** | | | | | | |
| North & South Thames | 135 | 34.6 | 49 | 31.4 | 78 | 37.7 |
| Trent, West Midlands, Anglia & Oxford | 105 | 26.9 | 42 | 26.9 | 49 | 23.7 |
| North & Yorkshire, North West | 62 | 15.9 | 29 | 18.6 | 37 | 17.9 |
| South & West | 58 | 14.9 | 21 | 13.5 | 32 | 15.5 |
| Wales | 17 | 4.4 | 1 | 0.6 | 5 | 2.4 |
| Scotland | 13 | 3.3 | 14 | 9.0 | 6 | 2.9 |
| **Total** | **390** | **100.0** | **156** | **100.0** | **207** | **100.0** |
| *Missing* | *21* | | *6* | | *10* | |

research or clinical trials ($P < 0.05$) and diet and nutrition ($P < 0.001$); there were also significant differences in requests about specific treatments, i.e. chemotherapy, hormonal therapy and radiotherapy ($P < 0.001$).

Among patients with colorectal cancer, men requested significantly less information about complementary therapies ($P < 0.05$) and had more concerns about prognosis ($P < 0.05$) than women. Men also requested emotional support and reassurance and information about treatment side effects less frequently than women, but these did not reach significance.

The socio-demographic characteristics of the sub-groups of prostate, and male or female colorectal cancer patients, who enquired about the four most frequent subjects, were not significantly different from all prostate, male or female colorectal cancer patients. However, more employed male colorectal cancer patients needed emotional support than expected, compared with all male colorectal cancer patients who contacted the service at this period (SER = 1.34, 95% CI: 0.97–1.81).

### Predictors of the four most frequent subjects of enquiry

The initial logistic regressions, taking all patients together, regardless primary site and gender, showed that primary site was a significant predictor ($P = 0.078$) for site specific information; gender was a significant predictor ($P = 0.015$) for emotional support/narratives. Primary site was a significant predictor ($P = 0.079$) for publications when gender remained at the model.

### Prostate cancer patients (Table 3)

Compared with retired prostate cancer patients aged 70 years or older, employed prostate cancer patients below 59 years old were less likely, while employed and aged 60 years or older men were more likely to request emotional support. Men less than 59 years of age were significantly more likely to ask for publications, but were less likely to enquire about specific therapies than older men.

### Colorectal cancer patients (Table 4)

The initial regressions for both male and female colorectal cancer patients showed that the interaction of gender with employment status was very significant ($P = 0.000$) for predicting emotional support/narratives. The interaction of gender with geographic location had an overall effect ($P = 0.137$) to publication requests. The interaction of gender with social class had an overall effect ($P = 0.159$) to specific therapies group.

The above results become more significant when we perform the analyses for males and females separately. Employed men with colorectal cancer were less likely to enquire about site-specific information, but were more likely to request emotional support than retired men. Patients from all other geographical areas were

**Table 2** Frequent subjects of enquiry from prostate, male and female colorectal cancer patients

| The most frequent subjects of enquiry | Prostate cancer patients | | Male colorectal cancer patients | | Female colorectal cancer patients | | Prostate patients versus male colorectal Significance* | Male colorectal versus female colorectal Significance* |
|---|---|---|---|---|---|---|---|---|
| | *n* = 411 | (%) | *n* = 162 | (%) | *n* = 217 | (%) | | |
| Site specific information[1] | **140** | **(34.1)** | **43** | **(26.5)** | **64** | **(29.5)** | | |
| Emotional support / narratives[2] | **154** | **(37.5)** | **66** | **(40.7)** | **104** | **(47.9)** | | |
| Emotional support and reassurance | 137 | (33.3) | 57 | (35.2) | 96 | (44.2) | | |
| Narratives and catharsis | 17 | (4.1) | 9 | (5.6) | 8 | (3.7) | | |
| Publications / booklist[3] | **133** | **(32.4)** | **65** | **(40.1)** | **76** | **(35.0)** | | |
| Specific therapy enquiries group[4] | **196** | **(47.7)** | **80** | **(49.4)** | **104** | **(47.9)** | | |
| Chemotherapy | 11 | (2.7) | 55 | (34.0) | 72 | (33.2) | $P < 0.001$ | |
| Complementary or alternative therapies | 5 | (1.2) | 4 | (2.5) | 16 | (7.4) | | $P < 0.05$ |
| Hormonal therapy | 125 | (30.4) | 0 | | 0 | | $P < 0.001$ | |
| Radiotherapy | 123 | (29.9) | 13 | (8.0) | 16 | (7.4) | $P < 0.001$ | |
| Surgery | 69 | (16.8) | 24 | (14.8) | 34 | (15.7) | | |
| Treatment enquiries | | | | | | | | |
| General treatment enquiry | 39 | (9.5) | 10 | (6.2) | 5 | (2.3) | | |
| Treatment side effects | 89 | (21.7) | 34 | (21.0) | 60 | (27.6) | | |
| Research or clinical trials | 15 | (3.6) | 14 | (8.6) | 11 | (5.1) | $P < 0.05$ | |
| Treatment centres or doctors | 19 | (4.6) | 7 | (4.3) | 11 | (5.1) | | |
| Other medical enquiries | | | | | | | | |
| Clarification of information | 65 | (15.8) | 21 | (13.0) | 34 | (15.7) | | |
| Diet and nutrition | 11 | (2.7) | 15 | (9.3) | 35 | (16.1) | $P < 0.001$ | |
| Prognosis | 37 | (9.0) | 19 | (11.7) | 10 | (4.6) | | $P < 0.05$ |
| Recurrence | 30 | (7.3) | 11 | (6.8) | 16 | (7.4) | | |
| Symptom control | 31 | (7.5) | 10 | (6.2) | 17 | (7.8) | | |
| Other support | | | | | | | | |
| Health professional communications | 47 | (11.4) | 16 | (9.9) | 23 | (10.6) | | |
| Sexuality and sexual problems | 21 | (5.1) | 3 | (1.9) | 1 | (0.5) | | |

Enquirers could ask about a number of different issues. The nurses could code up to 6 subjects of enquiry for every user. Only the most frequent subjects enquired are presented. Numbers do not add up to the total number of enquirers. [1] 'Site specific information' related to queries for information about a particular cancer, e.g. questions like 'what is prostate cancer?' 'how does it develop?'. [2]Enquiries that required emotional support and reassurance, e.g. queries like 'how can I cope?', or related to narratives or catharsis, e.g. enquirers who wanted to talk and mainly unload themselves, were grouped together to form 'Emotional support/narratives'. [3]'Publications' represented any requests for Cancer BACUP booklets, fact sheets and booklists. [4]Queries about at least one specific cancer treatment (Chemotherapy, complementary or alternative therapies, hormonal therapy, immunotherapy, radiotherapy, surgery and any other cancer treatment), e.g. 'what does chemotherapy involve', were grouped together to form 'Specific therapy enquiries'. *The Pearson and Likelihood ratio Chi-squares for independence have been calculated with Yate's correction. 2-sided Exact Significance is recorded, when $P = < 0.05$.

more likely to request publications than those from Thames. Patients in manual classes were less likely to enquire about specific therapies than those in non-manual classes.

Like their male counterparts, employed women were more likely to request emotional support than retired women, and women from most other regions were more likely to request publications than those from Thames. However, in contrast to men, the most significant predictor about site-specific information enquiries was geographic location; women from Trent, West Midlands, Anglia and Oxford were less likely to enquire about site-specific information than women from Thames (odds ratio: 0.36, 95% CI: 0.15–0.88).

### DISCUSSION

The sample of patients used in this study is representative of those patients contacting the service, but it is not representative of all cancer patients. Other common cancer sites, e.g. lung and Non-Hodgkin's lymphoma, have not been examined because of small sample numbers. Despite the number of people using the CancerBACUP Information Service, the small numbers of patients examined led to wide confidence intervals, may have resulted in a small number of significant variables, reduced significance levels (Altman et al, 2000) and reduced power of the goodness of fit test (Garson, 2000).

A previous analysis of first time users – patients, relatives and friends – of the information service between April 1995 and March 1996 revealed that, compared with the incidence of these cancers, there were more enquiries about prostate cancer (SIR: 1.15) and fewer enquiries about colorectal cancer (SIR for males: 0.89, SIR for females: 0.49) than expected (Boudioni et al, 1999a). The median ages of patients enquired about and the enquiry rates of unemployed and manual classes were lower than expected; enquiry rates from South and Central England were higher than expected (Boudioni et al, 1999a). The present analysis of patients using the service during the 2 following years, shows similar distributions of socio-demographic characteristics (Table 1), though enquiries from relatives and friends were excluded and a different methodology was used.

This study demonstrates that there are both similarities and differences in the information and support requests between men with different types of cancer (prostate and colorectal), and between men and women with the same type of cancer (colorectal). Interestingly, similar information was requested most frequently from all those patients (Table 2), perhaps reflecting common domains of information and support needs. The National

 *© 2001 Cancer Research Campaign*

BJOC 01-1945 641-648 20/8/01 3:11 pm Page 645

**Table 3** Prostate cancer patients – predictors of the four most frequent subjects of enquiry (*n* = 411, Logistic regressions based on 353 cases with complete data)

| **Commonest subjects**<br>**Significant predictors** | | **Adjusted odds ratio** | **95% CI** | ***P* value**** |
|---|---|---|---|---|
| **Site specific information***** | ***n* = 140** | | | |
| Social class | | | | |
| Non-Manual (reference category) | 82 | 1.00 | | 0.167 |
| Manual | 25 | 0.71 | (0.40 to 1.26) | |
| Unclassified | 21 | 0.59 | (0.32 to 1.09) | |
| Emotional support / narratives | ***n* = 154** | | | |
| Age of patient and employment status* | | | | |
| < 59 and employed | 10 | 0.46 | (0.20 to 1.04) | |
| 60–69 and employed | 35 | 1.89 | (0.98 to 3.63) | |
| 70 + and employed | 13 | 1.84 | (0.74 to 4.55) | |
| 60–69 and retired | 40 | 0.67 | (0.38 to 1.18) | |
| 70 + and retired (reference category) | 40 | 1.00 | | 0.001 |
| **Publications** | ***n* = 133** | | | |
| Age of patient | | | | |
| < 59 | 27 | 2.14 | (1.09 to 4.17) | |
| 60–69 | 55 | 0.88 | (0.53 to 1.46) | |
| 70 + (reference category) | 40 | 1.00 | | 0.021 |
| **Specific therapy enquiries** | ***n* = 196** | | | |
| Age of patient | | | | |
| < 59 | 20 | 0.49 | (0.25 to 0.96) | |
| 60–69 | 100 | 1.01 | (0.64 to 1.61) | |
| 70 + (reference category) | 64 | 1.00 | | 0.062 |

The variables entered into the logistic regression model were patient's age, employment status, social class and geographic location. The final model produced by backward elimination with the Likelihood-Ratio Criterion. *'< 59 and retired' category has been excluded because there were not any prostate cancer patients enquiring about emotional support/narratives in this category. **Significance of

Cancer Alliance study (1996) also reported that most men were as keen to obtain adequate information about their condition and treatment as women.

Among men, the significant differences in the rates of their enquiries about treatments and research or clinical trials may reflect the different treatments used for prostate and colorectal cancers and the research activities around them. Similarly, the treatment morbidity of colorectal cancer patients, including diarrhoea and constipation, may account for their increased need for diet and nutrition information.

Some of the differences between men and women reflect common gender stereotypes. Women's more frequent use of information services (Greenglass, 1992; Manfredi et al, 1993; Boudioni et al, 1999a; Green and Pope, 1999; Williams et al, 1999) may indicate a willingness to 'explore' alternative avenues of enquiry; they may be more open to non-conventional treatment, such as complementary therapies. Men are more interested in practical issues (Moynihan, 1998), which may explain their increased need for prognostic information. Though the occurrence of distress is similar for men and women (Fuhrer et al, 1999) women talk about their problems more openly (Harrison et al, 1995). Female colorectal cancer patients, in particular, have been found to report more emotional distress than males (Northouse et al, 2000). These findings are reflected by women in this study more frequently requesting emotional support.

This study has also identified key factors which are predictive of requests for information and support in relation to particular subjects (Tables 3 and 4). Perhaps most striking is the relationship between employment status and requests for emotional support from all patients (Tables 3 and 4). These findings highlight the impact, in Western society, of employment on the emotional needs of cancer patients (with the possible exception of younger men with prostate cancer) (Moynihan, 1996, 1998), regardless of gender, and lend support to the 'job model', rather than to the 'gender model' (Emslie et al, 1999). This is supported by the gender segregation of the labour market (Hunt and Annandale, 1999) reflected in our sample (Table 1). For men with cancer, in particular, the loss of a job can have devastating effects, both financially and psychologically (Kirby and Kirby, 1999), and the desire to get well and 'return to normal' may be expressed in terms of a desire to return to work (Seidler, 1989, 1998).

Among prostate cancer patients, age was a determinant of the kind of information or support sought (Table 3). Older men have been reported as more likely to feel 'helpless and hopeless' than younger men (Akechi et al, 1998), and may therefore need more emotional support. Older men are likely to be aware of prostate cancer (MORI, 1999), although few feel that sufficient information has been directed specifically to them (Health Education Authority, 1996). This may explain why they were less likely than younger men to request general information such as publications, but more likely to request specific therapies' information. Younger men may want to hide behind a 'brave façade' (Moynihan, 1998) and asking for written information may be easier than requesting emotional support or even information on specific therapies.

The importance of area of residence in shaping requests for publications amongst both male and female colorectal cancer patients (Table 4) may reflect regional differences in services, inequalities in NHS resource allocation or inaccessibility of health care services (Hart, 1997; The Stationery Office, 1998).

The observation that people from lower social classes make less effective use of health services (Office of Population, 1990) has

*© 2001 Cancer Research Campaign* 

**Table 4** Colorectal cancer patients – predictors of the four most frequent subjects of enquiry from males – Comparison with females

| Male patients *n* = 162, Logistic regressions based on 142 cases with complete data | | | | Female patients *n* = 217, Logistic regressions based on 187 cases with complete data | | | |
|---|---|---|---|---|---|---|---|
| **Commonest subjects Significant predictors** | **Adjusted odds ratio** | **95% CI odds ratio** | ***P* value*** | **Commonest subjects** | **Adjusted** | **95% CI** | ***P* value*** |
| **Site specific information** | ***n* = 43** | | | **Site specific information** | ***n* = 64** | | |
| Employment status | | | | Employment status | | | |
| Employed (reference category) | 16 | 1.00 | 0.071 | Employed (reference category) | 24 | 1.00 | 0.868 |
| Retired and other | 22 | 2.02 (0.94 to 4.35) | | Retired | 24 | 1.14 (0.48 to 2.68) | |
| | | | | Unemployed and other | 13 | 0.89 (0.40 to 2.00) | |
| **Emotional support / narratives** | ***n* = 66** | | | **Emotional support / narratives** | ***n* = 104** | | |
| Employment status | | | | Employment status | | | |
| Employed (reference category) | 43 | 1.00 | 0.000 | Employed (reference category) | 43 | 1.00 | 0.046 |
| Retired and other | 17 | 0.26 (0.12 to 0.53) | | Retired | 23 | 0.44 (0.19 to 1.01) | |
| | | | | Unemployed and Other | 27 | 1.26 (0.60 to 2.63) | |
| **Publications** | ***n* = 65** | | | **Publications**** | ***n* = 76** | | |
| NHS Health Authority | | | | NHS Health Authority | | | |
| N & S Thames (reference category) | 16 | 1.00 | 0.071 | N & S Thames (reference category) | 23 | 1.00 | 0.042 |
| Trent, West Midlands, Anglia & Oxford | 20 | 2.70 (1.06 to 6.87) | | Trent, West Midlands, Anglia & Oxford | 14 | 0.98 (0.43 to 2.24) | |
| North and Yorkshire, North West | 12 | 1.88 (0.66 to 5.33) | | North and Yorkshire, North West | 12 | 1.26 (0.51 to 3.11) | |
| South and West | 8 | 2.18 (0.70 to 6.89) | | South and West | 17 | 2.94 (1.21 to 7.19) | |
| Scotland | 6 | 5.40 (1.49 to 19.59) | | Scotland | 4 | 5.71 (0.96 to 33.86) | |
| **Specific therapy enquiries** | ***n* = 80** | | | **Specific therapy enquiries***** | ***n* = 104** | | |
| Social class | | | | Social class | | | |
| Non-manual (reference category) | 51 | 1.00 | 0.071 | Non-manual (reference category) | 55 | 1.00 | 0.519 |
| Manual | 10 | 0.38 (0.16 to 0.90) | | Manual | 8 | 0.71 (0.27 to 1.84) | |
| Unclassified | 13 | 0.96 (0.37 to 2.44) | | Unclassified | 31 | 1.27 (0.67 to 2.43) | |

**Male patients**: The variables entered into the logistic regression model for enquiries from male patients were patient's age, employment status, social class and geographic location. Wales were excluded from the logistic regression analysis for colorectal cancer. The final model produced by backward elimination with the Likelihood-Ratio Criterion. *Significance of Log Likelihood Ratio statistic, if term removed.

**Female patients**: For comparison, only the significant predictors for males were fitted into a simple enter logistic regression model for females, adjusted for age. The results are presented. *Significance of Log Likelihood Ratio statistic, if term removed. **This variable was also the last variable to remain when a multivariate backward logistic regression model was fitted. ***This variable was also the last variable removed from the model when a multivariate backward logistic regression model was fitted.

*© 2001 Cancer Research Campaign*

BJOC 01-1945 641-648 20/8/01 3:11 pm Page 647

also been noted in this study. Again, this may reflect unequal access to information services (Manfredi et al, 1993; Harris, 1998). The lower rate of enquiry for specific treatments from male colorectal cancer patients in manual classes (Table 4) may signify a mismatch of informational needs and/or ways that information is communicated (Wynne, 1992). There may be a disinclination among certain groups to become involved with particular aspects of information (Wynne, 1992), and in this case cancer management (Van Der Mollen, 1999; Leydon et al, 2000).

In summary, this study has demonstrated the complexity that underlies the information and support seeking behaviour of male cancer patients. No single pattern of information or support seeking was found among all male patients, nor were men's requests consistently different from women's requests. No single factor was found to predict the most frequent requests; on the contrary various factors affected the requests and there were both similarities and differences by site and gender. Further research will be needed to enable a better understanding of:

- How age affects prostate cancer patients' needs for information and support.
- Colorectal cancer patients' use of health services.
- Other cancer patients and family/carers' use of health services.
- Employment issues and the effect of cancer on employment and practical/financial issues.
- Inequalities in accessibility of services and delivery of information from manual classes and people from specific regions.

The further development of information and support services for men will need to take cognisance of their overall poor take-up of existing services, different take up by men with different illness and socio-demographic characteristics (Boudioni et al, 1999a, 1999b) and of other factors that may shape the specific needs of the individual. In another study, we found, for example, that more men living alone contacted the service than those in the general population (Boudioni et al, 1999b). The use of services and some information and support requests may be shaped by the cancer site's incidence and morbidity, which deserve special consideration, as in some cases demand may surpass capacity, while in others demand is low. Delivery and development of services should be flexible and respond to requests across a wide range of subjects in a way that is sensitive to the specific needs of the individual.

### ACKNOWLEDGEMENTS

This study was funded by the Cancer Research Campaign/ Educational and Psychosocial Research Committee. We thank all the CancerBACUP Information Service nurses and those who helped with the data checking and entry during the study period. We thank Alison Jones, Jean King and Elizabeth Wilson for their contribution. We also thank Ingrid Schoon and Brigitte Raimbault for their technical support.

### *Conflict of interest*

CancerBACUP provides information and support to those affected by cancer.

### REFERENCES

Akechi T, Okamura H, Yamawaki S and Uchitomi Y (1998) Predictors of patients' mental adjustment to cancer: patient characteristics and social support. *BJC* **77**: 2381–2385

Altman DG (1997) Relation between several variables. In: *Practical statistics for medical research*, Altman DG (ed) pp 325–364. Chapman and Hall: London

Altman DG, Gore SM, Gardner MJ and Pocock SJ (2000) Statistical guidelines and checklists. In: *Statistics with confidence*, Altman DG, Machin D, Bryant TN, Gardner MJ (eds) pp 171–201. BMJ Books: Bristol

Audit Commission (1993) *What Seems to be the Matter: Communication between Hospitals and Patients*. NHS Report No 12, HMSO: London

Boudioni M, McPherson K, Mossman J, Boulton M, Jones AL, King J, Wilson E and Slevin ML (1999a) An analysis of first-time enquirers to the CancerBACUP information service: variations with cancer site, demographic status and geographical location. *BJC* **79**: 138–145

Boudioni M, Mossman J, Jones A, Boulton M, Leydon G, McPherson K and Wilson E (1999b) Do enquirers to CancerBACUP Information Service have support at home? Poster presentation, *Cancer Research Campaign Grantees Meeting*: London

Department of Health (1992) *On the State of the Public Health: the annual report of the Chief Medical Officer of the Department of Health for the year 1992*. HMSO: London

Department of Health (1997) *The new NHS, Modern-Dependable*. DoH: London

Department of Health (1998) *A first class service-Quality in the new NHS*. DoH: London

Department of Health (1999) *No place in the NHS for inequalities of care*. NICE Press Release: London

Department of Health (2000a) *The NHS Cancer Plan. A Plan for Investement. A Plan for reform*. DoH: London

Department of Health (2000b) *£ 1M cash boost for prostate cancer research*. DoH Press Release 2000/0127: London

Emslie C, Hunt K and Macintyre S (1999) Problematizing gender, work and health: the relationship between gender, occupational grade, working conditions and minor morbidity in full-time bank employees. *Soc Sci Med* **48**: 33–48

Expert Advisory Group on cancer to the chief medical officers of England and Wales (1995) *A policy framework for commissioning cancer services. Guidance for purchasers and providers of cancer services*. DoH: London

Fallowfield L, Ford S and Lewis S (1995) No news is not good news: information preferences of patients with cancer. *Psycho-Oncology* **4**: 197–202

Fuhrer R, Stansfeld SA, Chemali J and Shipley MJ (1999) Gender, social relations and mental health: prospective findings from an occupational cohort. *Soc Sci Med* **48**: 77–87

Garson GD (2000) Logistic Regression. In: *PA 765 Statnotes: An Online Textbook: A guide to writing empirical papers, theses and dissertations*, Garson GD (ed). Available from: www2.chass.ncsu.edu/garson/pa765/logistic.htm (accessed April 2000)

Green CA and Pope CR (1999) Gender, psychosocial factors and the use of medical services: a longitudinal analysis. *Soc Sci Med* **48**: 1363–1372

Greenglass ER (1992) A gender-role perspective of social support: implications for psychological functioning. *Int J Psychol* **27**: 601

Harris K (1998) The informational needs of patients with cancer and their families. *Cancer Practice* **6**: 39–46

Harrison J, Maquire P and Pitcearthy C (1995) Confiding in crisis: gender differences in pattern of confiding among cancer patients. *Soc Sci Med* **41**: 1255–1260

Hart N (1997) Social inequalities in health. In: *The Sociology of Health and Medicine*, Hart N (ed) pp 50–77. Causeway Press: Lancashire

Health Education Authority (1996) *Men's attitudes to health checks and awareness of male-specific cancers*. Health Education Authority: London

Hunt K and Annandale E (1999) Relocating gender and morbidity: examining men's and women's health in contemporary Western societies. Introduction to Special Issue on Gender and Health. *Soc Sci Med* **48**: 1–5

Kirby RS and Kirby MG (1999) Men's health: closing the gender gap. In: *Men's health*, Kirby RS, Kirby MG, Farah RN (eds) pp 1–3. ISIS Medical Media: Oxford

Leydon GM, Boulton M, Moynihan C, Jones A, Mossman J, Boudioni M and McPherson K (2000) Cancer patients' information needs and information seeking behaviour: in depth interview study. *BMJ* **320**: 909–913

Manfredi C, Czaja R, Price J, Buis M and Janiszewski R (1993) Cancer patients' search for information. Monographs, *J NCI* **14**: 93–104

Men's Health Forum (1997) *Men's Health Forum: 1997 Strategy & Report*. Men's Health Forum: London

MORI social research (1999) *Men's Health; Awareness of testicular and prostate cancer; Research study conducted for the Institute of Cancer Research's Everyman Campaign*. MORI: London

Moynihan C (1996) Psychosocial assessments and counselling of the patient with testicular cancer. In: *Testicular cancer: Investigation and management*, Horwich A (ed) pp 403–419. Chapman & Hall: London

© 2001 Cancer Research Campaign 

BJOC 01-1945 641-648 20/8/01 3:11 pm Page 648

Moynihan C (1998) Theories in health care and research: Theories of masculinity. *BMJ* **317**: 1072–1075

National Cancer Alliance (1996) *"Patient-centred cancer services?" what patients say*. National Cancer Alliance: Oxford

Northouse LL, Mood D, Templin T, Mellon S and George T (2000) Couples' patterns of adjustment to colon cancer. *Soc Sci Med* **50**: 271–284

Office for National Statistics (2000) *Cancer statistics registrations; Registrations of cancer diagnosed in 1994, England & Wales*. Series MB1, no 27, The Stationery Office: London Office of Population, Censuses and Surveys (1990) *1971–1983 Longitudinal study: Socio-demographic differences in cancer survival*. Kogevinas E (ed), HMSO: London

Seidler V (1989) Identity. In: *Rediscovering masculinity: Reason, Language and Sexuality*, Seidler V (ed) pp 107–122. Routledge: New York

Seidler V (1998) Men, bodies, illness and vulnerability. Abstracts for ESPO 10, *Psycho-oncology* **7**: 165

Slevin ML, Terry Y, Hallett N, Jefferies S, Launder S, Plant R et al (1988) BACUP-the first two years: evaluation of a national cancer information service. *BMJ* **297**: 669–672

The Stationery Office (1998) *Our healthier nation, a contract for health (A consultation paper)*. The Stationery Office: London

The Stationery Office (2000) *The NHS Plan. A plan for investment. A plan for Van reform*. The Stationery Office: London

Van Der Mollen (1997)

Williams ERL, Ramirez AJ, Richards MA, Young T, Maher EJ, Boudioni M and Maquire P (1999) Are men missing from cancer information and support services? Abstract of poster presentation; *16th Annual Conference of the British Psychosocial Oncology Society*: London

Wynne B (1992) Misunderstood misunderstanding: social identities and public uptake of science. *Public Und Sci* **1**: 281–304

 *© 2001 Cancer Research Campaign*
